# Learning efficient haptic shape exploration with a rigid tactile sensor array

**DOI:** 10.1371/journal.pone.0226880

**Published:** 2020-01-02

**Authors:** Sascha Fleer, Alexandra Moringen, Roberta L. Klatzky, Helge Ritter

**Affiliations:** 1 Neuroinformatics Group, Bielefeld University, Bielefeld, Germany; 2 Department of Psychology, Carnegie Mellon University, Pittsburgh, Pennsylvania, United States of America; Politechnika Krakowska im Tadeusza Kosciuszki, POLAND

## Abstract

Haptic exploration is a key skill for both robots and humans to discriminate and handle unknown objects or to recognize familiar objects. Its active nature is evident in humans who from early on reliably acquire sophisticated sensory-motor capabilities for active exploratory touch and directed manual exploration that associates surfaces and object properties with their spatial locations. This is in stark contrast to robotics. In this field, the relative lack of good real-world interaction models—along with very restricted sensors and a scarcity of suitable training data to leverage machine learning methods—has so far rendered haptic exploration a largely underdeveloped skill. In robot vision however, deep learning approaches and an abundance of available training data have triggered huge advances. In the present work, we connect recent advances in *recurrent models of visual attention* with previous insights about the organisation of human haptic search behavior, *exploratory procedures* and *haptic glances* for a novel architecture that learns a generative model of haptic exploration in a simulated three-dimensional environment. This environment contains a set of rigid static objects representing a selection of one-dimensional local shape features embedded in a 3D space: an edge, a flat and a convex surface. The proposed algorithm simultaneously optimizes main perception-action loop components: feature extraction, integration of features over time, and the control strategy, while continuously acquiring data online. Inspired by the *Recurrent Attention Model*, we formalize the target task of haptic object identification in a reinforcement learning framework and reward the learner in the case of success only. We perform a multi-module neural network training, including a feature extractor and a recurrent neural network module aiding pose control for storing and combining sequential sensory data. The resulting haptic meta-controller for the rigid 16 × 16 tactile sensor array moving in a physics-driven simulation environment, called the *Haptic Attention Model*, performs a sequence of haptic glances, and outputs corresponding force measurements. The resulting method has been successfully tested with four different objects. It achieved results close to 100% while performing object contour exploration that has been optimized for its own sensor morphology.

## Introduction

While the sense of touch is central to human life, tactile capabilities of robots are currently hardly developed. This stark contrast becomes even more apparent if one compares touch and vision: while good camera sensors have become affordable and ubiquitous items and huge image and video databases together with deep learning have brought computer vision close (some would argue on par) to human vision [1–3], comparable advances in robot touch are widely lacking [4–7].

One reason is the very limited maturity of tactile sensors as compared with human skin. A second and deeper reason is that touch differs from vision in an important way: while looking at an object leaves its state unaffected, touch requires physical contact, coupling the sensor and the object in potentially complex and rich ways that usually also change the position, orientation or even the shape of the object. Human haptics makes active and sophisticated use of this richness to lend us skills such as haptic exploration, discrimination, manipulation and more. Large parts of these tasks are hard or impossible to model sufficiently accurately to replicate them on robots, thereby calling again for machine learning approaches similar to those that were highly successful in vision. However, the highly interactive nature of touch makes not only the learning problem itself much more difficult but also creates a problem for the availability of meaningful training data, since information about interactive haptics is much harder to capture in databases of static tactile patterns. As a consequence, learning approaches for the modality of interactive touch are still largely in their infancy and tactile skills enabling robots to establish and control rich and safe contact with objects or even humans are still a largely unsolved challenge which severely limits the use of robots in both domestic and industrial applications.

In this work we focus on using machine learning for the synthesis of one central and important haptic skill: the discrimination of unknown object shapes through a sequence of actively controlled haptic contacts between a sensor and the object surface. Our approach builds on recent advances that show how a deep network can be made to learn to integrate a sequence of visual observations to discriminate visual patterns. We extend this approach from the visual to the haptic domain and—by taking inspiration from insights about the organization of haptical exploration in humans—we create a potentially interesting new bridge between a computational understanding of interactive touch in robotics and in human haptics.

In humans, haptic capabilities are available at birth, for example, those that are necessary for a neonate to nurse. Over the course of early development, increasingly sophisticated haptic exploration comes on-line, as children acquire motor control and the ability to focus attention. By pre-school age, children demonstrate adult-like patterns of exploration [8] that they gate according to contextual demands [9]. This developmental process results in a small set of optimized action patterns, widely known under the term *exploratory procedures* (EPs) [10]. Humans use EPs to extract properties such as texture, hardness, weight, volume, or local shape features.

Under some circumstances, the level of complexity in haptic exploration can be effectively reduced to what was termed the *haptic glance* by Klatzky and Ledermann [11]. Specifically, they define the haptic glance as brief, spatially constrained contact that involves little or no movement of the fingers. In the same work they pose the question how the information from a haptic glance is translated into effective manipulation. Following this work, we are interested in a connection/transition between a haptic glance and an exploratory procedure. We propose that a haptic glance constitutes an atomic, primitive exploratory entity. We furthermore assume that an EP can be represented by a sequence of such primitives, if parameterization of each individual haptic glance is chosen in an optimal way. On a long-term scale, we are targeting the question: How can one model optimal control of haptic glances for optimal task-specific haptic exploration of an unknown object or scene? Will the resulting sequence of haptic glances emerge as a full EP? In order to answer this question affirmatively, such a control model should ideally contain a strategy to efficiently extract task-specific cues based on previously available information (if any), and integrate these over time. For computational purposes we make the following assumptions. Firstly, we assume that a haptic glance—being the simplest haptically directed action—is a foundation for any more complex haptic behavior, including haptic exploratory procedures of any type. Therefore, it is our goal to learn an optimal sequence of haptic glances, adapted to a given task and a sensor morphology that is provided beforehand and is specific for a given robot platform. Secondly, we assume that a haptic glance is defined by a tuple consisting of a pressure profile yielded by the tactile sensor at contact and the corresponding sensor pose.

Robots, like humans, benefit from haptic sensors in order to find, identify, and manipulate objects. Tactile sensing applications in robotics are built out of two different categories [12]. The first one is called “perception for action”, which utilizes the tactile information to solve dexterous manipulation tasks including grasping, slip prevention. The second category, which has recently become a popular area of research, is named “action for perception”, dealing with recognition and exploration [13–15]. Recent developments have added machine learning techniques in order to learn exploration strategies, feature extraction or a better estimation of different quantities. One class of methods is *reinforcement learning*, a biologically inspired class of learning methods in which the agent learns by gathering data through the active exploring of the environment [16]. It is applied to teach a robot dexterous manipulation [17, 18] or to use learned exploration strategies in the form of tactile skills in order to facilitate exploration as studied for surface classification [15].

The approach employed in this work provides one possible solution to a typically puzzling question: how to couple optimization of both above-mentioned directions, “perception for action”, and “action for perception”. In computer vision, the analogous question has already been investigated by measures of *recurrent models of visual attention* (RAM) [19, 20]. RAM acquires image glimpses by controlling the movement of a simulated eye within the image. The modeling approach is inspired by the fact that humans are not perceiving their environment as a whole image. Instead, they see only parts of the scene, while the location of the fixations depends on the current task [21, 22]. The model gathers information about the environment directed by image-based and task-dependent saliency cues [23, 24]. Information extracted from these foveal “glimpses” is then combined in order to get an accumulated understanding of the visible scene. RAM applied to control of the sequences of haptic glances optimizes both above-mentioned directions simultaneously in a series of iterative steps, and enables us to find an optimal solution for a given tactile end-effector, with respect to its own constraints and the spatio-temporal resolution of the acquired data.

Inspired by this work, we present a framework that is able to identify four different objects using a tactile sensor array within a simulated environment. The object classification and pose control are formalized as a sequential decision-making process within a reinforcement learning framework, where an artificial agent is able to perform multiple haptic glances before the final estimation of the object’s class. During the training of a multi-component deep neural network, we learn how to control the pose of the rigid tactile sensor in a way that is beneficial for the classification task. To enable integration of information gained through multiple haptic glances, we employ a recurrent neural network as one building block of this architecture. The next section describes the simulation setup and the employed algorithm, together with the training procedure. After presenting the conducted experiments, we summarize and discuss the obtained results.

## Scenario and experimental setup

**Fig 1:**
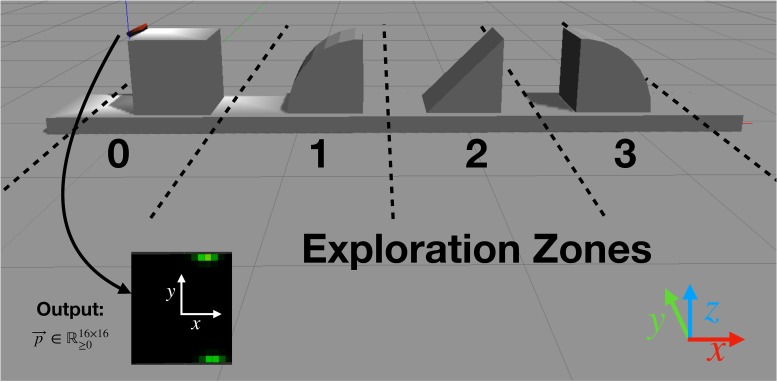
Gazebo simulation. The image displays a view of the linear object arrangement along the *x*-axis with four objects from 0 to 3 and the simulated Myrmex sensor (small red blob touching object 0). The bottom side of the sensor contains the square-shaped pressure sensitive tactile array, measuring a pressure profile on contact with the object that is visualized by the square on the bottom left of the image. Additionally, the borders of the *exploration zone* for each object are indicated by dotted lines.

To develop an efficient haptic controller that can enable a robot to identify objects with a sequence of haptic glances, we perform a comprehensive experimental investigation in Gazebo (see S1 Code), a physics-driven simulation environment. The simulation consists of two main parts as illustrated in Fig 1.

### The tactile sensor array—Myrmex

The first part is a floating standalone tactile sensor array, modeled to resemble the *Myrmex* [25] sensor in order to ease the transfer to a real robot in future experiments. It is constructed out of a circular end-effector mount (red) with a square sensitive zone (black). In simulation, one side of the sensor contains a square-shaped array of 16 × 16 cells covering a surface of 64 cm^2^, whose values are computed to approximately resemble the values of the real sensor array (see S2 Code). Contacts at collision are estimated by Gazebo’s physics engine ODE according to inter-penetration of objects (intrinsic compliance) and to default local surface parameters. An example of the contact information available in Gazebo and its characteristics are shown in S1 Video.

**Fig 2:**
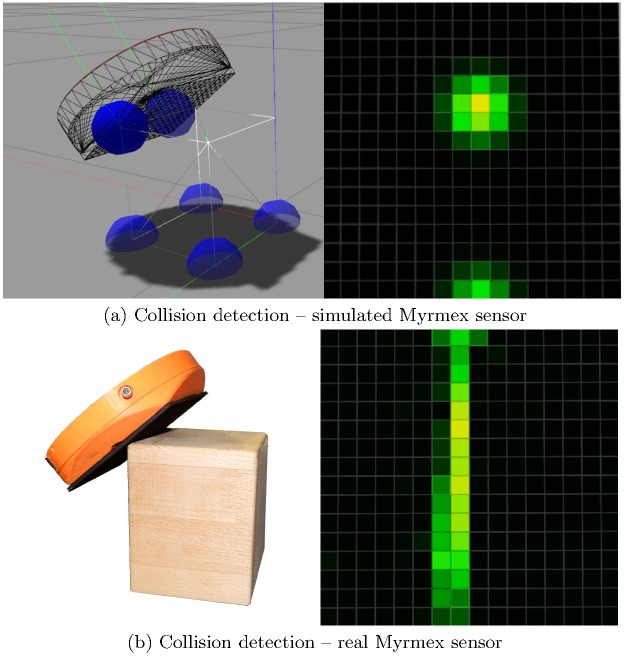
Comparison of the simulated and real sensor. A comparison of the measurements between simulated and real Myrmex sensor when contacting an object edge. The left side of (a) sketches the measuring process of the simulated Myrmex. Blue spheres illustrate the measured contact information leading to the tactile image on the right side of (a). Figure (b) shows the real sensor, together with the measured tactile image.

Each contact defined by its position and force vector, generates a Gaussian distribution around the contact center with amplitude depending only on the normal force. The standard deviation is arbitrarily fixed to mimic the deformation of the sensitive foam on the real sensor. Mixing the distributions creates a 16 × 16 tactile pressure image, that is represented as an array of floating point values contrary to the real sensor with only 4096 levels of pressure. When measuring the collision with an edge as it is illustrated in Fig 1, we expect to see a line. However, due to the limitations of the collision library, we acquire the image presented in the bottom left corner. In Fig 2 the tactile image for a contact with a cuboid is shown for both the simulated Myrmex sensor (Fig 2(a)), and the real sensor (Fig 2(b)). The collision library libccd used by the ODE simulation engine of Gazebo can generate only two contact points at a time (see S1 Video). Consequently, it is not possible to produce an edge in the resulting tactile image. On the contrary, the real sensor produces a tactile image in which the expected line of contact is visible.

Communication with the simulated sensor in Gazebo is performed via a ROS-interface (see S3 Code).

### Stimulus material

The second part is the stimulus material. It exists as a static set of 3D objects that are distributed in the simulation environment, but also in the form of real 3D wooden building blocks with 3D elementary shapes carved on top. Our current set of elementary shape types consists of approximately 60 prototypes. A combination of such building blocks forms the so-called Modular Haptic Stimulus Board (MHSB) (see S2 Video for design and applications and S1 Project for the MHSB project web-site). By rearranging the blocks, MHSBs of different sizes and different shape configurations have been previously employed in a range of studies of haptic exploration and search in humans (e.g., [26–28]). Through its modularity, MHSB enables a flexible experimental design resulting in a wide range of 3D shape landscapes.

All shapes within the current setup are rigid, stationary and have the same height. Building blocks employed for this experiment, 9 × 9 cm each, were chosen, firstly, to suit the size of the real Myrmex sensor and the restrictions of its control with the real KUKA robot arm. For this work, we have chosen a set of objects locally representing basic types of one-dimensional curvature features, e.g. edge, flat descendent/horizontal surface, and a convex surface. Due to the fact that concave surfaces may be more challenging for the simulated sensor, we are omitting them in the current work. This one-dimensional curvature design enabled us to constrain parameterization of haptic glances to two dimensions, translation and rotation along one axis, together with the linear arrangement of the shapes, without loss of generality. In case new features are considered within the experimental stimulus design, new types of control parameters as well as new output have to be used for an implementation of the haptic glance controller. For example, in case objects are equal w.r.t. the curvature and can be differentiated based on height only (a set of cuboids of different heights), a haptic glance controller needs to output the height of collision with the object as well as the pressure profile.

### Haptic control of the simulated Myrmex

Haptic control consists of two parts, a low-level controller that performs haptic glances and a higher-level controller that provides parameterization for the low-level controller and is responsible for solving the task.

#### The high-level meta-controller—HAM

The process of haptic exploration is operated by the so-called meta-controller: the *Haptic Attention Model* (HAM). It is represented by a deep neural network and is described in detail in the Methods Section. Its main task is to classify the given object, while constantly providing a new expedient target pose *ξ* = (*x*_*g*_, *y*_*g*_, *z*_*g*_, *e*_1_, *e*_2_, *e*_3_) of the sensor, including three position coordinates (*x*_*g*_, *y*_*g*_, *z*_*g*_) and three orientations (*e*_1_, *e*_2_, *e*_3_), to the low-level controller for further exploration. It performs the optimization for the parameterization of haptic glances based on the state of the networks’ working memory, a representation of the previously acquired haptic data. For proof of concept, we restricted the number of parameters that have to be provided by the HAM to the position along the *x*-axis and the angle around the *y*-axis. Before the execution of the haptic glance, the sensor is positioned at a specific pose where *x*_*g*_ and the Euler angle *e*_2_ are specified by the output of the network $\vec{l}={\left(x_{g},e_{2}\right)}^{⊤}$. For the sake of readability, the alterable position *x*_*g*_ is called *x* and the angle *e*_2_ is called *φ* in the following sections.

#### The low-level haptic glance controller

**Fig 3:**
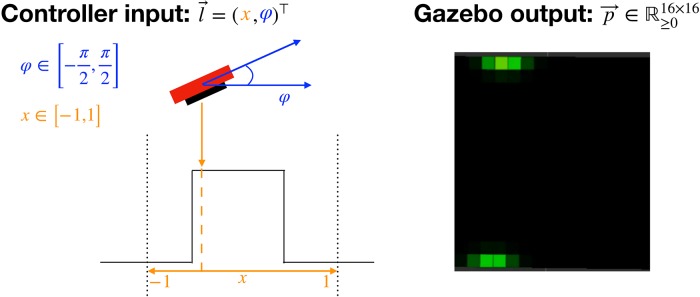
Schematic illustration of the experiment’s core idea and its realization in simulation. The experimental setup contains four objects whose positions are static. Myrmex gathers information about the objects by performing haptic glances at position x and orientation φ around the y-axis, leading to a 16 × 16 pressure image.

Without loss of generality, we use a simplified and naive representation of the low-level controller as illustrated in Fig 3. It executes a primitive haptic interaction specified by two parameters which are provided by the HAM. Given a pose, it outputs the acquired pressure, $g:\left(x,\varphi \right)\to \vec{p}$. An execution of the glance controller moves Myrmex from a predefined (*x*, *y*, *z*)-position down along the *z*-axis. To this end, it gradually decreases its height—indicated by the value *z*—while keeping both the orientation and the (*x*, *y*) position constant until a collision with an object takes place (see S3 Video). Upon collision with the object, handled by the physics engine with minimal penetration when the overall pressure level on the sensor reaches a certain threshold, the motion stops and the sensor outputs its readings. To compute it, the forces applied to the 16 × 16 sensor cells of the Myrmex sensor are summed up. The threshold value is reached, when an overall force of 2 N is distributed over the contact surface of the Myrmex, i.e. 2 N/64 cm^2^ = 312.5 Pa. The main feature of this controller, implemented with the “hand of god” plugin (see S4 Code), is the constant sustainment of the sensor’s orientation and the (*x*, *y*) position up to the time of collision. This is realized by switching off the gravity and continuously holding the sensor pose at a predefined value against the impact of any impulses. By this means, the full control of both the pose parameters and the resulting tactile measurement is guaranteed. Additionally, this restricted implementation resembles the movement of the sensor when attached to a robot arm.

In this work, determined by the type of tactile sensing available as well as the restricted design of the haptic object properties, the haptic glance controller employed by the network is parameterized only by the pose. However, the parameterization may be extended, or, alternatively, a set of differently parameterized haptic glance controllers, similar to a functional basis, may be employed by the network. An example of an extension would be a function $\hat{g}:\left(x,\varphi \right)\to \left(\vec{p},h\right)$ that maps from the pose to the tuple containing both the pressure and the corresponding height. Such parameterization is necessary in case the stimuli differ in height. If we further extend the shape complexity from the one-dimensional to a two-dimensional curvature feature, two orientation parameters instead of one will account for the data acquisition, i.e. $\hat{g}:\left(x,\varphi_{x},\varphi_{y}\right)\to \left(\vec{p},h\right)$.

### Classification task

During training and classification, the agent is always presented with one out of four objects. It explores the restricted object space with the sensor by performing a predefined number of haptic glances. In order to learn an exploration policy that is independent of the object’s pose within the global coordinate system, we introduce *exploration zones* illustrated with dashed lines in Fig 1. Exploration zones are pre-defined regions with their own local coordinate systems, in which the objects are placed for exploration. After specification of the exploration zone, two out of six pose parameters of the tactile sensor can be modified by the high-level meta-controller as explained in the previous section. To preclude learning the absolute position of the object, its coordinates within the simulation space are mapped to an exploration zone *x* ∈ [−1, 1], corresponding to the range in which the output of the neural network lies. Due to the location of the pressure-sensitive surface on only one side of the Myrmex, rotations are performed within the range *φ* ∈ [−*π*/2, + *π*/2]. Further rotation will not yield contact information between the object and the sensor surface. The acquired pressure information is employed not only to classify the given object but also to determine the next position and orientation of the sensor in the next exploration step.

## Methods

Reinforcement learning is a well-known class of machine learning algorithms for solving sequential decision-making problems through maximization of a cumulative scalar-valued reward function [16]. To formalize our task as a reinforcement learning problem, the artificial agent receives a reward of *r* = 1 for a correctly classified object and a reward of *r* = 0 otherwise. We then use the standard formulation of a Markov decision process defined by the tuple (*S*, *A*, *P*^*A*^, *R*, *γ*, *S*_0_), where *S* denotes the set of states and *A* the set of admissible actions. *P*^*A*^ is the set of transition matrices, one for each action *a* ∈ *A* with matrix elements Ps→,s→s′a specifying the probability to end up in state ${\vec{s}}^{\prime }$ after taking action *a* from state $\vec{s}$. Finally, r∈R⊂R is a scalar valued reward the agent receives after ending up in s→s′, *γ* the discount factor and *S*_0_ ⊆ *S* is the set of starting states. The goal is to find an optimal policy *π*: *S* → *A* that maximizes the discounted future reward

$$\begin{array}{c} R_{t}=\sum_{k=0}^{\infty }\gamma^{k}r_{t+k}. \end{array}$$ (1)

The discount factor *γ* ∈ [0, 1) balances the weighting between present rewards and rewards that lie increasingly in the future.

A neural network with a set of weights *θ* can be employed to solve a reinforcement learning task, i.e., its output should maximize a given reward function *R*_*t*_. In this case we can perform a gradient-based policy optimization with the help of the REINFORCE update rule [29, 30]. The general rule for updating the corresponding weights *θ* of the network is thus given by

$$\begin{array}{c} \Delta_{\theta }=\alpha \cdot [r_{t}-b\left({\vec{s}}_{t};\theta \right)]\cdot \zeta \left({\vec{s}}_{t};\theta \right), \end{array}$$ (2)

where *α* defines the learning rate factor, *b* the reinforcement baseline. *ζ* is called the *characteristic eligibility*. It is defined as

$$\begin{array}{c} \zeta \left({\vec{s}}_{t};\theta \right)=\frac{\partial \log f({\vec{s}}_{t};\theta )}{\partial \theta }, \end{array}$$

where $f({\vec{s}}_{t};\theta )$ determines the trainable output of the network as a function of its input ${\vec{s}}_{t}$ and its weight parameters *θ*. Using REINFORCE, it is thus possible to develop learning rules for stochastic policies that depend on multiple input parameters, like an adaptable Gaussian with variable mean *μ* and standard deviation *σ*. To this end, a neural network is trained to map the input to a parameterization of the Gaussian distribution, i.e., *μ* and *σ*. Instead of their corresponding weights *θ*_*μ*_ and *θ*_*σ*_, *μ* and *σ* themselves can be treated as the adaptable parameters of the Gaussian N(x;μ,σ). Using this simplification, the characteristic eligibility for *μ* is given by

$$\begin{array}{c} \zeta_{\mu }=\frac{\partial \log N(x;\mu ,\sigma )}{\partial \mu }=\frac{x-\mu }{\sigma^{2}}, \end{array}$$ (3)

where *x* is the corresponding value, sampled from the Gaussian distribution N. Analogously, the characteristic eligibility for *σ* is

$$\begin{array}{c} \zeta_{\sigma }=\frac{\partial \log N(x;\mu ,\sigma )}{\partial \sigma }=\frac{{\left(x-\mu \right)}^{2}-\sigma^{2}}{\sigma^{3}}. \end{array}$$ (4)

The details of the application of these equations to our work is described in the section below.

### The haptic attention model

**Fig 4:**
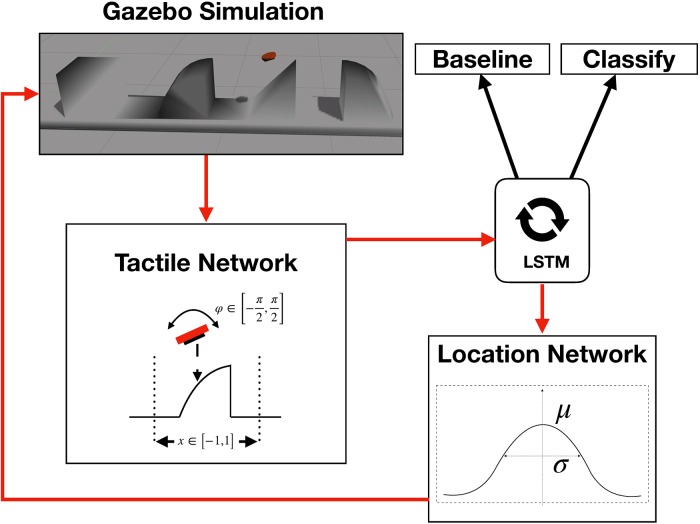
Illustration of the used model. The overall design of the multi-module meta-controller model and its interaction with the Gazebo simulation environment.

In the following, the architecture of our designed high-level meta-controller, called the *haptic attention model* is described in detail. An overview of the interaction loop between the network and the simulation is displayed in Fig 4. Inspired by the architectures in [19, 20], the meta-controller network is constructed from three modules which are described in detail in the following subsections (See also S5 Code). A vector $\vec{s}={\left(x,\varphi ,\vec{p}\right)}^{⊤}$ consisting of the sensor pose (*x*, *φ*) and the corresponding pressure profile acquired by Myrmex performing a haptic glance in Gazebo is used as the sensory input for the network. The 16 × 16 pressure matrix is flattened to a normalized pressure vector $\vec{p}$ with $\text{dim}(\vec{p})=256$. For the normalization we employ the *L*2-norm. Apart from the considerations of numerical stability during network training (no small/large numbers and no large differences), the normalization is performed in order to get rid of artifacts in the data caused by the method chosen to perform a haptic glance in simulation. These artifacts are specific to moving the floating Myrmex towards an object at an unknown position in tiny discrete steps, which is likely to produce a different strength of signal depending on the distance between the sensor and the object in the last step prior to collision. Therefore, normalization is performed in order to achieve a comparable pressure profile for a given pose, independent of the force, whose absolute strength in this particular case is a simulation artifact.

First, the input is processed through the *tactile network*, which combines the recorded pressure profile $\vec{p}$ with its corresponding location *x* and orientation *φ* into one single feature vector. The features $\vec{s}$ are then propagated through a *long short-term memory* (LSTM) network [31]. This kind of neural network belongs to the class of “recurrent neural networks” which have the ability to store, combine and process sequential data. It is constructed using hidden states of 256 neurons. The LSTM provides features to the *object classifier* and to the *location network* that in turn provides a new pose. Although the classification of the object can be done within each glance, we usually refer to the classification result after the final glance.

If not stated otherwise, all layers are connected through *rectified linear units* (ReLu) [32] as activation functions. The linear layers of the whole model are all built out of 64 neurons. For more information about (recurrent) neural networks see e.g., [33].

#### The tactile network

**Fig 5:**
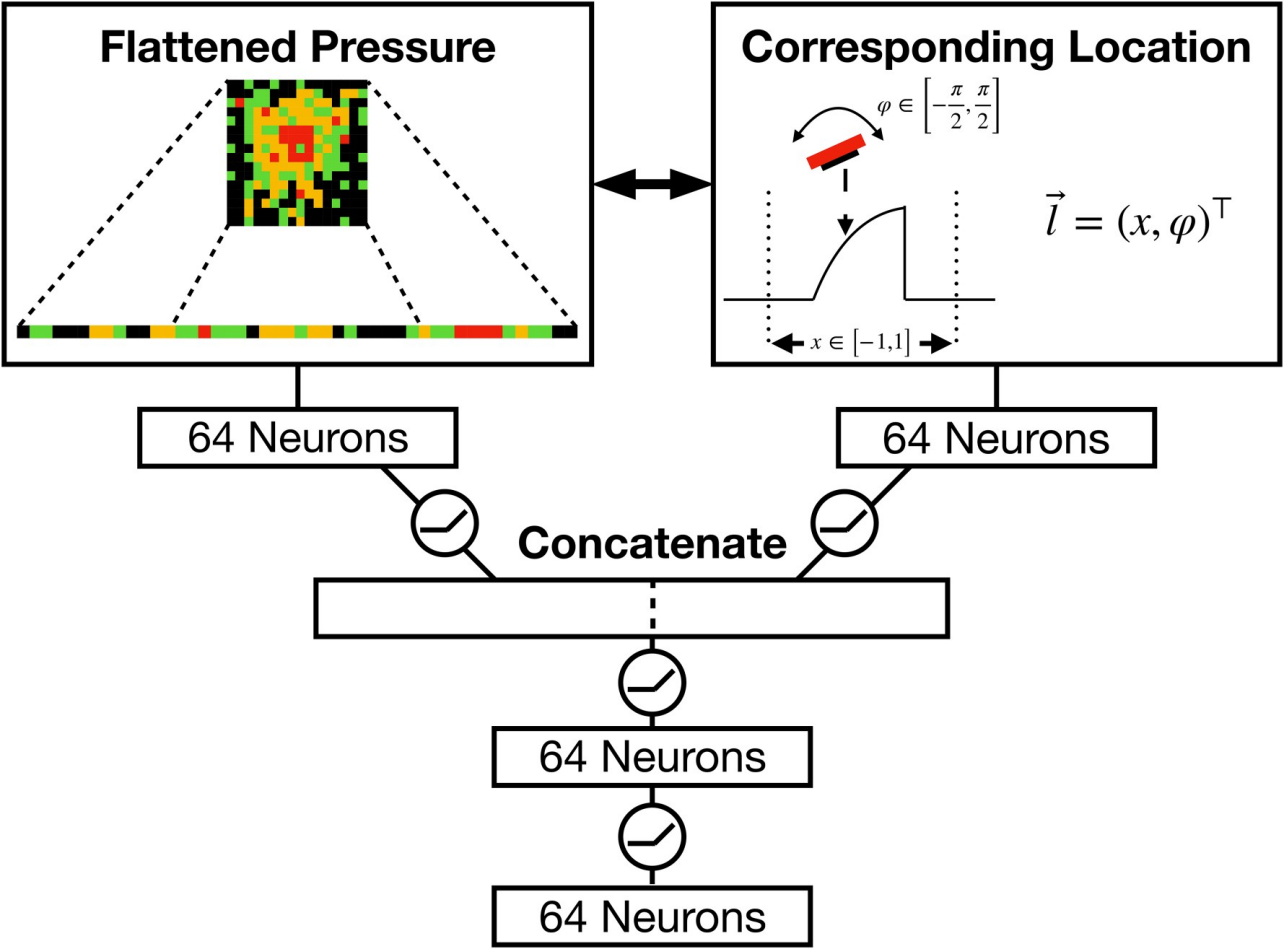
Illustration of the tactile network. The tactile network combines the normalized pressure $\vec{p}$ with the corresponding location *x* and orientation *φ*. Thus, the input vector for the pressure has the length $\text{dim}(\vec{p})=256$ and the input vector for the location-orientation pair $\text{dim}(\vec{l})=2$ respectively. The small circles in-between the connections indicate that the *ReLu unit* is used as the activation function.

The *tactile network* is displayed in detail in Fig 5. It combines the tactile response of the sensor $\vec{p}$ with the corresponding location *x* and angle *φ*. An important choice is the approach used to combine *what* (i.e., the pressure *p*) with *where* (i.e., position *x* and orientation *φ*). While [19] use an element-wise addition of the two features, [20, 34] suggest using element-wise multiplication. In this work, based on the performed tests, we concatenate the two resulting types of features followed by two additional linear layers. In this way, we do not impose a specific inner structure on the combination process, but let the network resolve this issue on its own.

#### The location network

**Fig 6:**
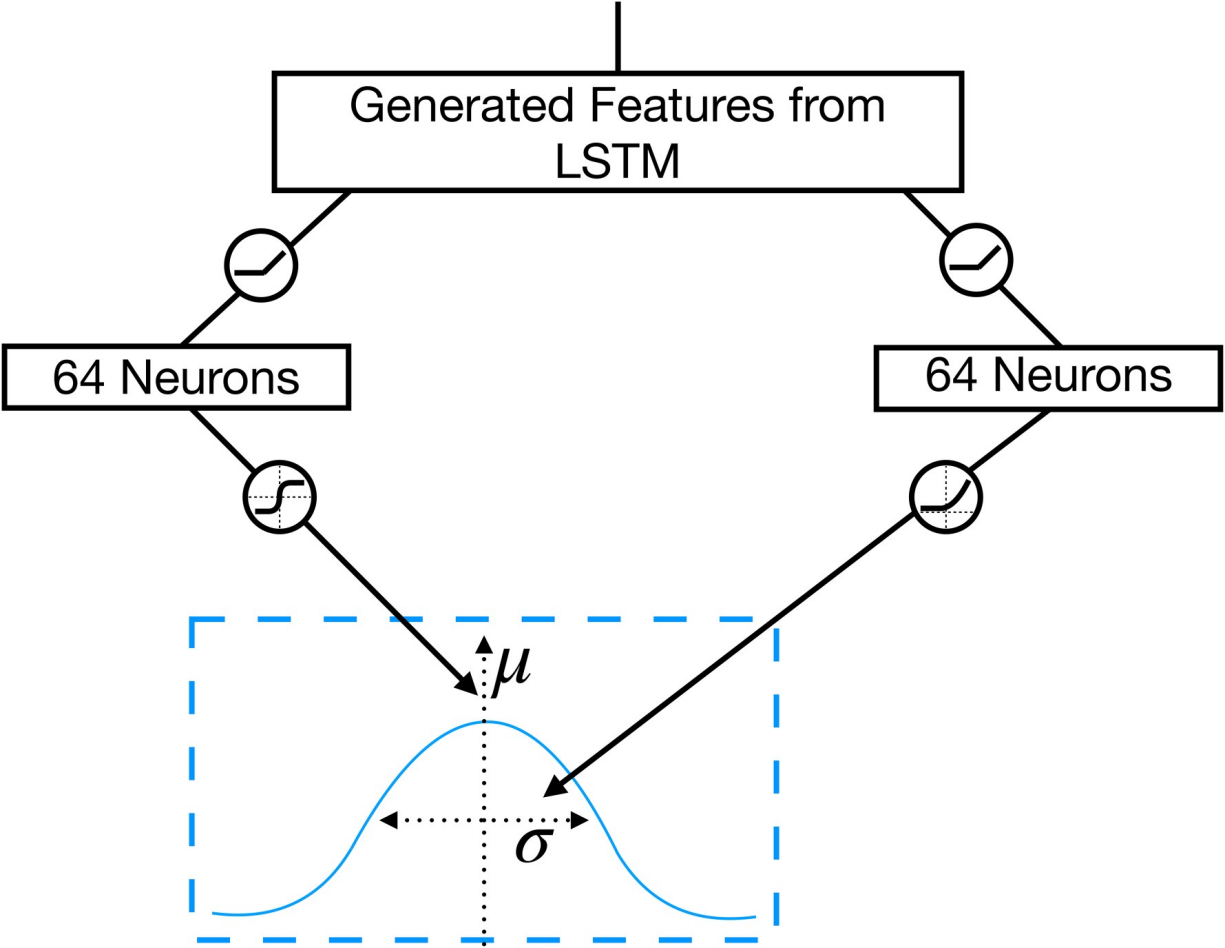
Illustration of the location network. The location network uses the generated features of the LSTM to determine a new location and orientation (mean *μ*, left branch) with the corresponding standard deviations (*σ*, right branch) for the tactile sensor using a stochastic policy.

The *location network* is designed to output the pose of the next haptic glance. The feature vector that is used as the input to this module is the output that is generated by the LSTM unit. It thus implicitly integrates shape information yielded by the previously performed glances. A stochastic location policy is modeled using two Gaussian distributions for position and orientation, respectively with variable mean *μ* and standard deviation *σ* as shown in Fig 6.

The features of the LSTM are propagated through a linear layer that outputs the mean *μ*(*θ*)∈[−1, 1] and the standard deviation *σ*(*θ*) of the Gaussian *θ* is referring to the corresponding weights of the model that are necessary to generate the desired output, which is in this case *μ* or *σ*. The extent of exploration of the location policy is given by the size of the Gaussian’s standard deviation *σ*. While for large *σ*, the raw location of the glance, given by *μ*, is imprecise, the location has more precision for smaller *σ*.

The two above-mentioned pipelines are used for computing a distinct *μ* and *σ* for the position and for orientation. The used activation function for the output layers are chosen to limit the resulting values to a reasonable range. While the *tanh* is used as the activation function to generate the mean within the desired range, the *softplus function* [35] is implemented as the activation function for the standard deviation. The output values *μ*(*θ*) and *σ*(*θ*) are then used to compute the new location and orientation by sampling from the respective 1-dimensional Gaussians for each of the desired variables.

To ensure that the location and position of the sensor remain within the predefined space around the to-be-classified object and also that the orientation remains within its boundaries, the sampled values of the Gaussians N(q;μ,σ) are again restricted to the range *q* ∈ [−1, 1]. Thus, if *q* is sampled outside this range, it is resampled. The new pose vector is then given as

$$\begin{array}{c} \vec{l}=(q_{x},q_{\varphi }\cdot \frac{\pi }{2})=(x,\varphi ). \end{array}$$

#### The classification network

In order to classify a given object, the generated feature vector of the LSTM is not only transferred to the *location network*, but also propagated through a different linear layer that is then used for classification. To achieve this, the *softmax-function* is utilized to encode the predicted class-affiliation of the current object *o* in a probability density $\pi (o|{\vec{\tau }}_{1:s};\theta_{t})$, representing the current policy of the reinforcement learning agent. Here, ${\vec{\tau }}_{1:S}\left(\theta_{t}\right)$ encodes the accumulated LSTM feature vector after *S* glances, using the current set of weights *θ*_*t*_ at training step *t*. For classification, the class *o* with the highest probability

$$\begin{array}{c} o=\underset{o^{\prime }}{\mathrm{argmax}}\pi \left(o^{\prime }|{\vec{\tau }}_{1:S};\theta_{t}\right) \end{array}$$ (5)

is taken as the prediction.

### Training

For each training step, a new batch of size 64 is generated, where the to-be-classified objects *o* are uniformly chosen from the set of all four available objects. The target loss function L, used for training, is composed of two different components: classification and location. The update rule for both parts is derived from the REINFORCE algorithm. For the classification component of the loss, we see the designed model as a reinforcement learner which has to choose the right action in order to classify the given object. For classifying the object correctly it receives a reward *r* = 1, and *r* = 0 otherwise. The predicted probability of correctly identifying the target object *o* after *S* glances is then given as $\pi (o|{\vec{\tau }}_{1:S};\theta )$. To this end, the *categorical cross-entropy* can be used to compute the loss.

For learning the means *μ*_*x*_ and *μ*_*φ*_ of the location component of the policy, the characteristic eligibility as outlined in Eq (3) is used. *σ*_*x*_ and *σ*_*φ*_ are learned by applying Eq (4). The hybrid update rule is then given by

$$\begin{array}{c} \Delta_{\Theta }=-\alpha \cdot [\beta \cdot \left(r_{t}-b_{t}\right)\cdot (\zeta_{\mu_{x}}+\zeta_{\mu_{\varphi }}+\zeta_{\sigma_{x}}+\zeta_{\sigma_{\varphi }})+\sum_{o=0}^{O}\log\left(\pi \left(o\right)\right)\cdot y_{o}]. \end{array}$$

The function *π*(*o*) gives the computed classification probability that the to-be-classified object is object *o*, while *y*_*o*_ is 1 if *o* corresponds to the correct object and 0 otherwise.

The parameter *β* controls the contribution of the different parts of the update. While for *β* = 1 both parts of the update contribute equally to the weight update, a smaller factor of *β* < 1 assigns more resources to the classification part. For *β* = 0, the part of the update that involves the location network is completely omitted [34].

The baseline layer is updated separately, using the mean-squared error. Instead of training the baseline only on the accumulated tactile information of the last glance ${\vec{\tau }}_{1:S}$, the training can be improved by also using all included sub-sequences ${\vec{\tau }}_{1:s}$ with *s* ≤ *S* [34]. This leads to the loss function

$$\begin{array}{c} L_{b}=\sum_{s=1}^{S}{\left[R_{s}-b\left({\vec{\tau }}_{1:s};\theta_{b}\right)\right]}^{2}. \end{array}$$ (6)

The overall network model is trained using stochastic gradient descent with Nesterov momentum [36, 37]. The chosen learning rate of *α*_0_ decays towards *α*_min_ every training-step *t* with a decay factor of *δ*_*α*_ and a step-size of *T* according to

$$\begin{array}{c} \alpha_{t}=\max(\alpha_{\text{min}},\alpha_{0}\cdot \delta_{\alpha }^{\frac{t}{T}}). \end{array}$$

Due to the design of the network that generates a location for the next haptic glance, no fixed training set can be used to train the classifier. The current batch specifies only the to-be-classified objects, while the first pressure-location pair is chosen by the first random glance for each object. The location for any further glance is chosen by the current state of the location policy of the network.

## Experiments

To perform an empirical examination of the validity of the network architecture, we perform a series of evaluations with a focus on each one of the three modules: the LSTM, the location network, and the tactile network. The core of the evaluation approach focuses on the recurrent LSTM unit that plays a central role in feature extraction and integration. Our hypothesis is that by employing LSTM we increase both the classification accuracy and the efficiency of the pose control. To test the efficiency of the LSTM on both tasks, the classification accuracy is computed while training the network on a varying number of glances. In addition to the final classification accuracy, the individual classification accuracies after each glance are evaluated. To demonstrate the efficiency of using a recurrent unit instead of a simple linear hidden layer, the experiment is repeated with the LSTM replaced by a linear layer of the same size (i.e., 256 neurons).

The second part of the evaluation is dedicated to the pose control by the location network. We evaluate it during the learning process, and compare the results against a model with a random location choice. To this end, we omit the location network and provide the model with new locations *x* ∈ [−1, 1] and orientations *φ* ∈ [−*π*/2, *π*/2] that are sampled from a uniform distribution. For training, only the classification part of Eq (6) is used to create the weight update, while *β* is set to 0.

In the third part of the evaluation, the different approaches for combining the tactile information with its corresponding location (What & Where) are compared.

All models are trained for 50 ⋅ 10^3^ steps. In order to measure the performance after a certain number of training steps, the training is stopped. This is followed by estimation of the mean classification accuracy of 100 newly generated batches, using the currently available policy. In our experiments, the “classification accuracy” or “classification performance” is defined as the probability of the model to correctly classify the current object. To obtain a statistically correct measure of the accuracy, each experiment is repeated 10 times. For the final evaluation, the mean accuracy of these experiments is computed with the standard deviation of the mean as the accuracy measure error.

### Hyperparameters

Table 1 lists the hyperparameters that are used for all experiments. The parameters are chosen according to *random search* [38] with a fixed number of 3 glances, followed by additional manual tuning. The weights of all layers are initialized using *He normal initialization* [39] with a bias of 0.

**Table 1:** Hyperparameters employed for network training.

Parameter	Value
Batch Size	64
Initial learning rate *α*	8 ⋅ 10^−4^
Learning rate decay factor *δ*_*α*_	0.97
Learning rate update step-size *T*	800
Minimal learning rate *α*_0_	10^−6^
Used optimizer	SGD with Nesterov momentum
Momentum	0.9
Location weight *β*	0.4
Hidden state size—LSTM	256 neurons
Size of linear layers	64 neurons

### Creation of the dataset

In order to perform quick optimization and testing, we conducted multiple experiments on a pre-recorded dataset $D_{o}$ (see S1 Dataset) generated in Gazebo, previous to the experimental runs, for each object *o*. The dataset contains tuples $d_{o}=\left(x,\varphi ,\vec{p}\right)$. Here $\vec{p}$ is the normalized pressure-vector $\vec{p}$, *x* ∈ [−1, 1] the respective position of the sensor within the location space and *φ* ∈ [−*π*/2, *π*/2] the angle. For each object the recording of the tuples *d*_*o*_ starts with the position *x* = −1 and the orientation *φ* = −*π*/2. These two parameters are then both incremented with a step size of Δ_*x*_ = 0.01 and Δ_*φ*_ = *π* ⋅ 0.01, leading to 201 × 201 prerecorded data-points *d*_*o*_ for each object. The complete dataset has then a size of roughly 161 ⋅ 10^3^ data-points that can be picked to approximate the sensor pose generated by the location network. For a new pair (*x*, *φ*) generated by the network, the closest data-point *d*_*o*_ is selected from the pre-recorded data set.

## Results

The main results of the conducted experiments are summarized in Table 2. It displays the classification accuracies for all three variants of the architecture as described above and shows the corresponding results for an increasing number of glances. The full meta-controller model $\pi_{M}$ contains all trained components including the LSTM module and the location network. The random location policy approach *π*_rloc_ substitutes the location network with a random location generator. *π*_MLP_ substitutes the LSTM unit with a linear layer of the same size with a ReLu as its activation function. As the neural network is now built out of linear layers only, it can be seen as a *multi-layer perceptron* (MLP). In the last column, labeled 〈*π*_MLP_〉, the classification performance of *π*_MLP_ is evaluated by averaging over all conducted glances.

**Table 2:** Best classification performance for the different number of glances.

# Glances	1. $\pi_{M}$	2. *π*_rloc_	3. *π*_MLP_	4. 〈*π*_MLP_〉
1	0.547 ± 0.002	0.547 ± 0.002	0.570 ± 0.003	0.570 ± 0.003
2	**0.831 ± 0.002**	0.753 ± 0.002	0.668 ± 0.003	0.645 ± 0.003
3	**0.910 ± 0.001**	0.858 ± 0.001	0.662 ± 0.003	0.829 ± 0.002
4	**0.942 ± 0.001**	0.917 ± 0.001	0.662 ± 0.003	0.912 ± 0.002
6	**0.977 ± 0.001**	0.971 ± 0.001	0.670 ± 0.004	0.976 ± 0.001
8	0.988 ± 0.001	0.990 ± 0.000	0.668 ± 0.004	0.993 ± 0.000
10	0.994 ± 0.001	0.995 ± 0.000	0.668 ± 0.003	0.997 ± 0.000

The table lists the best measured classification performance after 50 ⋅ 10^3^ training steps for the different tested model variants.

The “full model” $\pi_{M}$ (see column 1) reaches a classification accuracy of about 99.4% on the pre-recorded dataset. While the accuracy using one random glance is only ≈ 55%, it continuously improves when more glances can be executed. Granting the model just one more glance leads to an accuracy of about 83%. Overall, accuracy improvement for the full model is faster than for the other two tested architectures, up to its convergence after about 6 glances are performed.

Column 2 presents the results of the random location policy. It starts from the same performance as the full model (since the first glance is random in both policies) and from there approaches its asymptotic performance more slowly, making its performance inferior when only 2 to 6 glances can be invested. Thus, our model is able to learn to efficiently extract important information when the number of possible interactions with the given object are limited.

If the recurrent LSTM unit is replaced with a linear layer of the same size (column 3), the classification accuracy does not rise beyond 67%, constituting the worst result. Due to missing recurrent connection, and the fact that the accuracy is evaluated only after the last glance, the MLP-based architecture *π*_MLP_ is optimized based only on the last glance, and therefore does not improve after two glances.

However, by averaging its output according to

$$\begin{array}{c} o=\underset{o^{\prime }}{\mathrm{argmax}}\frac{1}{S}\sum_{s=1}^{S}\pi \left(o^{\prime }|{\vec{\tau }}_{s:S};\theta_{t}\right) \end{array}$$

the performance of this averaged MLP model becomes very similar to the random model (column 2). Asymptotically (here: ten or more glances), all except the MLP model reach practically perfect classification.

**Fig 7:**
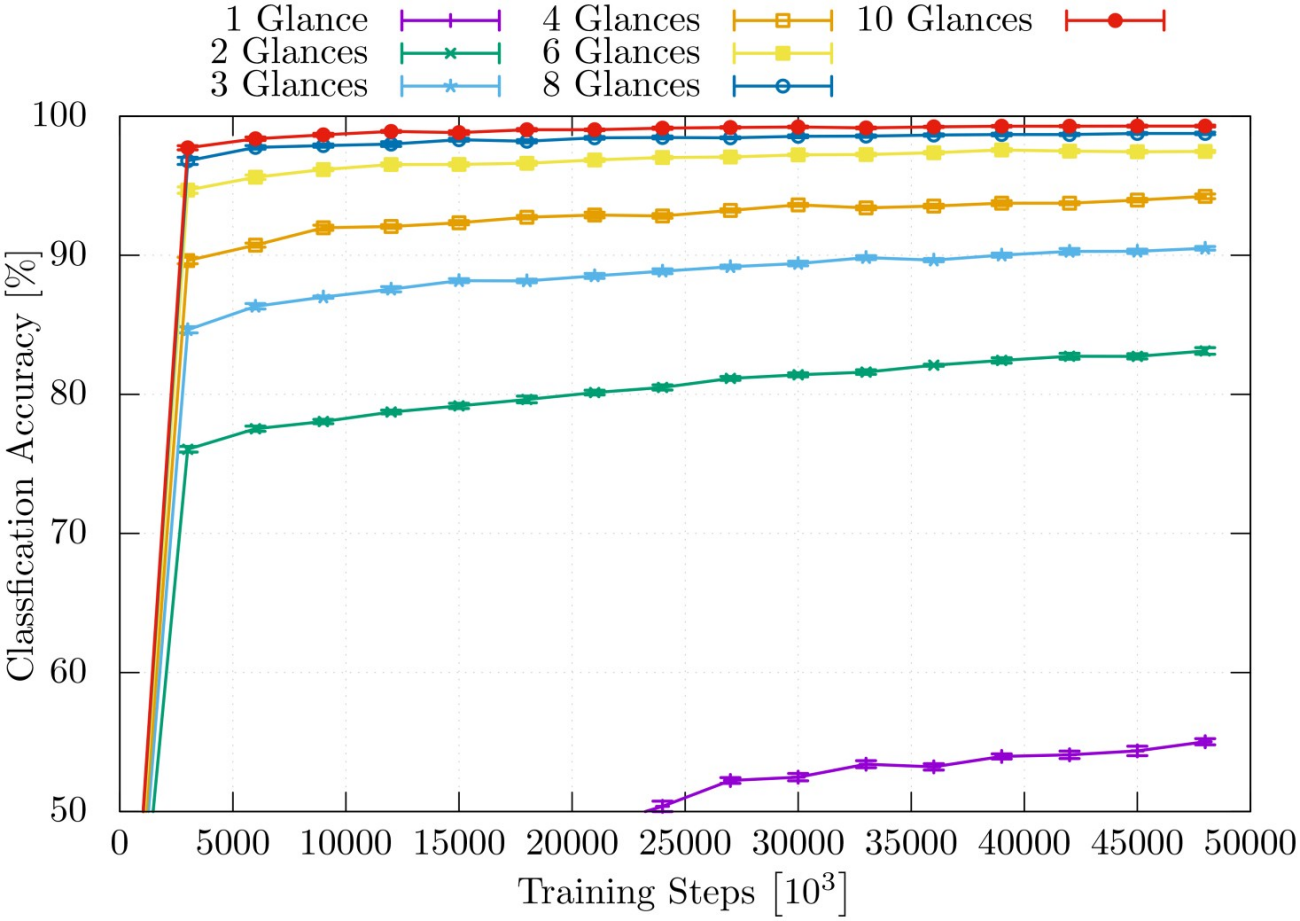
Classification accuracy. The time course of the classification accuracy during the training is visualized for the model while it is trained to classify using a different number of glances.

**Fig 8:**
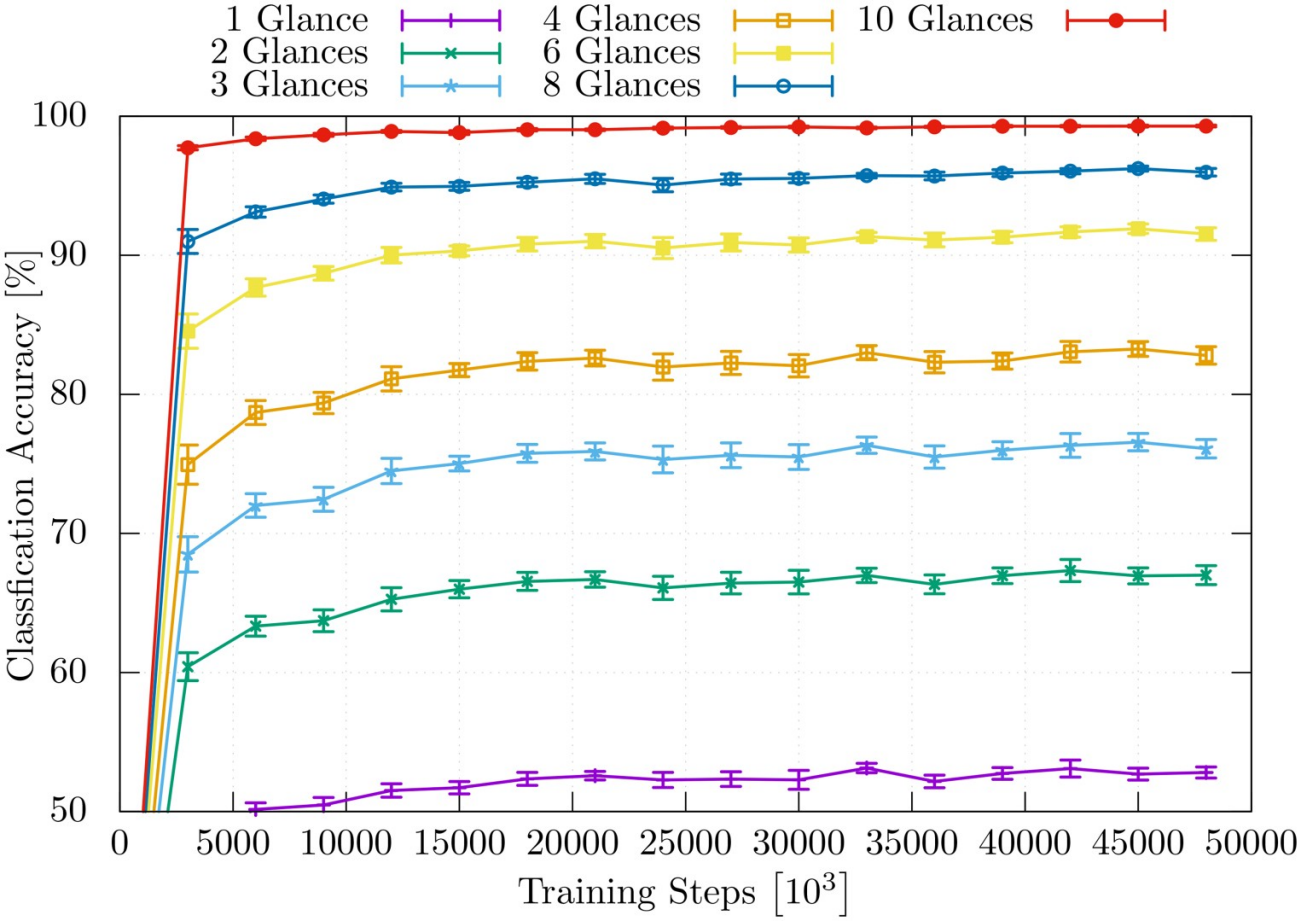
Classification accuracy. Classification accuracy of the individual glances in one classification event using the LSTM layer is displayed. The classification event uses 10 glances to classify each object.

Fig 7 shows the time course of learning of the model for the different numbers of performed glances. Additionally, the individual classification accuracy for each glance within one classification event that uses 10 glances is visualized in Fig 8. The accuracy of the individual glances within a classification event differs from the ones in Fig 7.

**Fig 9:**
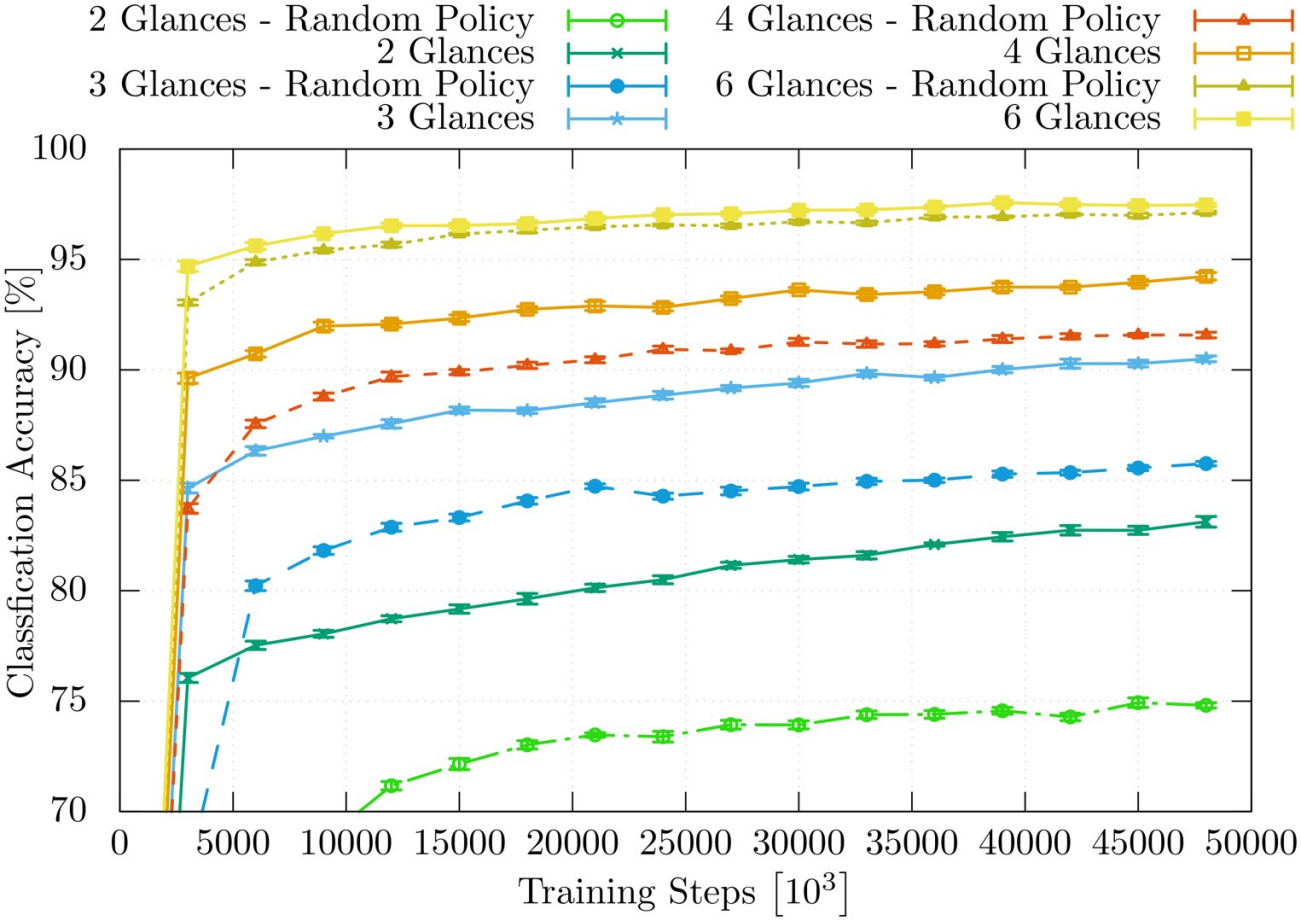
Classification accuracy. Comparison of classification accuracies between the model that relies on a learned location policy *π*_loc_ (solid lines) and the model that uses a random location policy (dashed lines).

Fig 9 presents a detailed performance comparison between the *π*_rloc_ and the full model $\pi_{M}$. Here, one can again see that a huge performance gap exists when the model is able to execute only a small number of glances and that this gap is progressively closed as the number of glances is increased. Fig 9 shows that the impact of the learned location policy is more visible when the model is trained on a smaller number of glances. The model $\pi_{M}$ learns to classify objects based on limited information more efficiently.

Table 3 lists the best classification accuracies of the model using 3 glances for the different ways of combining the normalized pressure vector $\vec{p}$ with the corresponding location $\vec{l}$. The procedure to combine the two sets of features via concatenation and then processing the result through two layers gives slightly better results than the element-wise addition, but clearly outperforms the approaches of element-wise multiplication and the concatenation approach using one layer.

**Table 3:** Learning performance: What & where.

Combiner	Best Performance
elem. multiplication	0.873 ± 0.002
concat. followed by 1 layer	0.899 ± 0.002
elem. addition	0.902 ± 0.001
**concat. followed by 2 layers**	**0.905 ± 0.001**

The table lists the best measured classification performance within the 50 ⋅ 10^3^ training steps for the different tested approaches of combining the normalized pressure $\vec{p}$ with the location-orientation pair (*x*, *φ*).

## Discussion

The performed evaluations demonstrated that the proposed model is able to classify the objects with an accuracy of nearly 100% by actively acquiring an optimized sequence of tactile sensor measurements. In this approach the data is generated on-the-fly by haptic interaction with the environment, performed by means of haptic glances and directed by the history of previous tactile events. The results of the conducted experiments show that the full network architecture $\pi_{M}$, including the recurrent LSTM network and the location network, is capable of controlling the execution of haptic glances in the most efficient way. The architecture performs better with a trained location network than with a random location policy *π*_rloc_. Employing the LSTM to represent the sequence history yields better performance in comparison to the memory-less architecture *π*_MLP_. Here, the location network only slightly improves the location w.r.t. the task-relevant information with the increasing number of glances. In comparison with $\pi_{M}$, a good but less efficient performance of the 〈*π*_MLP_〉 that accumulates individual classification decisions may be due to the averaging out of noise with the increasing number of glances. Therefore, both recurrence and an optimized location control are likely to be necessary ingredients of an efficient haptic exploration model in our scenario. These results may be constrained by the simplicity of the 3D shapes considered in the experiment. For an extensive evaluation of the proposed approach, the creation of data sets with a greater number of different objects would be necessary, including stimuli that are more challenging to differentiate without an optimized control strategy. For the described case, we expect that the efficiency and accuracy trends would become more evident.

The network architecture $\pi_{M}$ merely fuses and accumulates the data, whose representation is optimized with the goal to achieve the most accurate and efficient execution for a given task. Therefore, it provides a general interface, which has a capacity to accommodate for different types of haptic glance parameterizations. However, our approach to parameterization and control was deliberately very rudimentary in this work. The currently employed minimalistic haptic glance is controlled by a one-dimensional translation and rotation, characterized by a uni-variate pressure output. This simplification was coupled to the experimental design targeting exploration of one-dimensional curvature features. Other types of haptic glance controller parameterizations are desirable, in case other features than the curvature need to be explored. On the one hand, both the number, type of the control parameters and the outputs are very likely to be determined bottom-up by the features of the 3D environment in which haptic interaction is performed. On the other hand, they are determined in a top-down fashion by the task of the interaction. It remains an open question how to automatically derive the minimal parameterization and the output of the haptic glance controller depending on the features of the environment, the task, the available degrees of freedom of the employed device and its tactile capabilities.

The modularity of our model should, however, provide the functionality to adapt to more complex sensor devices as different modules of the HAM just have to be extended to cope with the increasing number of control dimensions. In the current configuration, our model has a total number of 741248 trainable weights. Within the simplest case, for each additional trainable parameter that is provided to the low-level haptic glance controller an additional output stream with at least one additional linear layer (with e.g. 64 neurons) has to be added to the location network. While this procedure does not necessarily lead to a significant increase in the number of trainable weights, too many additional control parameters might exceed the memory and processing capacity of the LSTM network. The LSTM network contains—in contrast to the linear layers—a large amount of the trainable weights. A necessary amplification of its size or the addition of a second LSTM network in order to increase performance might have a higher impact on the model’s size and its training time. While the designed hybrid loss might be a reasonable approach when only two control parameters have to be adapted, a higher number could slow down the convergence process of the model. One possible way out of this dilemma might be to separately train the classification and control part of the HAM with different loss functions that share the achieved reward.

## Conclusion and future work

In this work we have proposed the first implementation of a controller, inspired by the concept of *haptic glances*. Provided a pose parameter as an input, a floating tactile sensor array touches the surface at the specified location and yields the resulting pressure vector. We have trained a meta-controller network architecture to perform an efficient haptic exploration of 3D shapes by optimally parametrizing the haptic glance controller to perform a sequence of glances and identify 3D objects. Tests of the architecture have been successfully performed in a physics-driven simulation environment.

The structure of the meta-controller includes a mechanism that accumulates the data acquired during execution of the task and parameterizes the future haptic glances based on the optimized representation of this data. However, the current mechanism performing this temporal integration—based on an LSTM and inspired by the functionality of the working memory—may not be sufficient for an execution of a more complex task consisting of multiple task stages, such as e.g. haptic search, or a contact-rich object manipulation. In such tasks, it may be necessary to save the representation of data existing in the working memory to a long-term memory, from which this information could be retrieved at a later stage in the task execution. To this end, the meta-controller needs to communicate with an extra structure, based on e.g. hashing, such as Neural Turing Machine [40] to access features acquired at multiple previous time slots during interaction with the target topology.

To support our claim that the resulting policy can enable a robot equipped with a tactile sensor to perform efficient object identification by touch, we see performing tests with a (simulated) robot platform, equipped with a Myrmex tactile sensor array as our next task. Furthermore, we will extend our experimental design with the second curvature dimension and, corresponding to this, an extra degree of freedom in our haptic glance controller.

Due to the fact that the pose is sampled from a Gaussian distribution, it is highly unlikely that the same position or orientation is sustained during the exploration. Therefore, the current approach results in a jumpy energy-inefficient exploration trajectory which makes a more energy-efficient policy desirable. Consequently, the meta-controller optimization should be extended to enable a smoother trajectory generation. This may be possible by a careful shaping of the reward function or a further refinement of the location network.

Beyond performing haptic object identification, we believe that the developed procedure may be applied to enable a robot to perform complex manipulation tasks that heavily rely on haptics. Execution of a more complex tasks such as above-mentioned haptic search commonly involve multiple types of strategies, targeting exploration of different types of haptic features, e.g. movability or rigidity. This may be possible by implementing a set of low-level haptic glance controllers characterized by different parameterizations and outputs accompanied by a gating mechanism that enables the overall model to switch between them.

## Supporting information

S1 Code Gazebo. The simulation software is available under the following link: http://gazebosim.org/.
(DOCX)

S2 Code Myrmex simulation. Code of the tactile simulation is available under the following link: https://github.com/ubi-agni/gazebo_tactile_plugins.
(DOCX)

S3 Code ROS. The software is available under the following link: http://www.ros.org/.
(DOCX)

S4 Code The “hand of god” plugin. The plugin is available under the following link: https://github.com/ros-simulation/gazebo_ros_pkgs/blob/kinetic-devel/gazebo_plugins/src/gazebo_ros_hand_of_god.cpp.
(DOCX)

S5 Code The haptic attention model. A link to the source code can be found at: http://doi.org/10.4119/unibi/2936475.
(DOCX)

S1 Video Contact information in Gazebo. A visualization of the contact information in Gazebo.
(MP4)

S2 Video Modular Haptic Stimulus Board (MHSB). The video presents design and applications with MHSB https://www.youtube.com/watch?v=CftpCCrIAuw.
(DOCX)

S3 Video Gazebo simulation—Haptic glance controller. The video describes the experimental setting and illustrates the functionality of the haptic glance controller during object exploration.
(MP4)

S1 Project Modular Haptic Stimulus Board (MHSB). Project web-site is available under the following link: https://ni.www.techfak.uni-bielefeld.de/node/3574.
(DOCX)

S1 Dataset Recorded dataset of glance locations and the corresponding pressure data. The recorded dataset is available at: http://doi.org/10.4119/unibi/2936475.
(DOCX)
