# Correction: Learning efficient haptic shape exploration with a rigid tactile sensor array

**DOI:** 10.1371/journal.pone.0230054

**Published:** 2020-02-27

**Authors:** Sascha Fleer, Alexandra Moringen, Roberta L Klatzky, Helge Ritter

There are errors in the Funding statement. The correct Funding statement is as follows: This research/work was supported by the Cluster of Excellence Cognitive Interaction Technology 'CITEC' (EXC 277) at Bielefeld University to SF and AM, which is funded by the German Research Foundation (DFG).
