# Supplementary material for: Learning efficient haptic shape exploration with a rigid tactile sensor array
Source: PLoS One. 2020 Jan 2;15(1):e0226880. doi: 10.1371/journal.pone.0226880 (PMC6940144; doi:10.1371/journal.pone.0226880)
Supplement: S1 Code — The simulation software is available under the following link: http://gazebosim.org/. (DOCX) [file pone.0226880.s001.docx]

**S1 Code. Gazebo.** The simulation software is available under the following link: http://gazebosim.org/
