# Supplementary material for: Learning efficient haptic shape exploration with a rigid tactile sensor array
Source: PLoS One. 2020 Jan 2;15(1):e0226880. doi: 10.1371/journal.pone.0226880 (PMC6940144; doi:10.1371/journal.pone.0226880)
Supplement: S2 Code — Code of the tactile simulation is available under the following link: https://github.com/ubi-agni/gazebo_tactile_plugins. (DOCX) [file pone.0226880.s002.docx]

**S2 Code. Myrmex Simulation.** Code of the tactile simulation is available under the following link: https://github.com/ubi-agni/gazebo_tactile_plugins
