# Supplementary material for: Learning efficient haptic shape exploration with a rigid tactile sensor array
Source: PLoS One. 2020 Jan 2;15(1):e0226880. doi: 10.1371/journal.pone.0226880 (PMC6940144; doi:10.1371/journal.pone.0226880)
Supplement: S3 Code — The software is available under the following link: http://www.ros.org/. (DOCX) [file pone.0226880.s003.docx]

**S3 Code. ROS**. The software is available under the following link: http://www.ros.org/
