# Supplementary material for: Learning efficient haptic shape exploration with a rigid tactile sensor array
Source: PLoS One. 2020 Jan 2;15(1):e0226880. doi: 10.1371/journal.pone.0226880 (PMC6940144; doi:10.1371/journal.pone.0226880)
Supplement: S4 Code — The plugin is available under the following link: https://github.com/ros-simulation/gazebo_ros_pkgs/blob/kinetic-devel/gazebo_plugins/src/gazebo_ros_hand_of_god.cpp. (DOCX) [file pone.0226880.s004.docx]

**S4 Code. The “Hand of God” Plugin.** The plugin is available under the following link: https://github.com/ros-simulation/gazebo_ros_pkgs/blob/kinetic-devel/gazebo_plugins/src/gazebo_ros_hand_of_god.cpp
