# Supplementary material for: Learning efficient haptic shape exploration with a rigid tactile sensor array
Source: PLoS One. 2020 Jan 2;15(1):e0226880. doi: 10.1371/journal.pone.0226880 (PMC6940144; doi:10.1371/journal.pone.0226880)
Supplement: S2 Video — The video presents design and applications with MHSB https://www.youtube.com/watch?v=CftpCCrIAuw. (DOCX) [file pone.0226880.s007.docx]

**S2 Video. Modular Haptic Stimulus Board (MHSB).** The video presents design and applications with MHSB: https://www.youtube.com/watch?v=CftpCCrIAuw
