# Supplementary material for: Learning efficient haptic shape exploration with a rigid tactile sensor array
Source: PLoS One. 2020 Jan 2;15(1):e0226880. doi: 10.1371/journal.pone.0226880 (PMC6940144; doi:10.1371/journal.pone.0226880)
Supplement: S1 Project — Project web-site is available under the following link: https://ni.www.techfak.uni-bielefeld.de/node/3574. (DOCX) [file pone.0226880.s009.docx]

**S1 Project. Modular Haptic Stimulus Board (MHSB).** Project web-site is available under the following link: https://ni.www.techfak.uni-bielefeld.de/node/3574
