# Supplementary material for: Learning efficient haptic shape exploration with a rigid tactile sensor array
Source: PLoS One. 2020 Jan 2;15(1):e0226880. doi: 10.1371/journal.pone.0226880 (PMC6940144; doi:10.1371/journal.pone.0226880)
Supplement: S1 Dataset — The recorded dataset is available at: http://doi.org/10.4119/unibi/2936475. (DOCX) [file pone.0226880.s010.docx]

**S1 Dataset. Recorded Dataset of Glance Locations and the Corresponding Pressure Data.** The recorded dataset is available at: http://doi.org/10.4119/unibi/2936475
